# Effects of Combined Treatment With Selective Androgen and Estrogen Receptor Modulators Ostarine and Raloxifen on Bone Tissue In Ovariectomized Rats

**DOI:** 10.1007/s00223-025-01431-4

**Published:** 2025-10-24

**Authors:** Daniel B. Hoffmann, Marius Staub, Kai O. Böker, Arndt F. Schilling, Paul J. Roch, Wolfgang Lehmann, Stefan Taudien, Swantje Oberthür, Stephan Sehmisch, Marina Komrakova

**Affiliations:** 1 Department of Trauma, Orthopaedic- and Plastic Surgery, Georg-August-University of Goettingen, Robert Koch St. No. 40, 37075 Goettingen, Germany; 2 Department of Medical Microbiology, Subdivision of General Hygiene and Environmental Health, University of Goettingen, Humboldallee 34a, 37073 Goettingen, Germany; 3 Department of Trauma Surgery, Hannover Medical School, University of Hannover, Carl-Neuberg-Str. 1, 30625 Hannover, Germany

**Keywords:** Raloxifen, Ostarine, SARM, SERM, Osteoporosis, Ovariectomized rat model

## Abstract

Hormone replacement therapy in women usually focuses on estrogen and progesterone replacement. But also androgens decrease with age in women. Decrease of estrogen or androgens levels are associated with bone loss, while steroid hormone therapy often leads to negative side effects. A possible solution could be the use of selective receptor modulators (SRMs). Here, a combined treatment with a selective estrogen receptor modulator (raloxifene, RAL) and a selective androgen receptor modulator (ostarine, OST) was investigated in ovariectomized rats. The study was performed using 3-month-old female Sprague–Dawley rats. Fifteen control rats were not ovariectomized (NON-OVX). Sixty rats were ovariectomized and divided into 4 groups (n = 15/group): (1) untreated rats (OVX), (2) rats receiving OST (0.55 ± 0.08 mg/kg body weight [BW]), (3) rats receiving RAL (11.07 ± 1.77 mg/kg BW), (4) rats treated with OST and RAL. The compounds were administered orally as osteoporosis prophylaxis immediately after ovariectomy for up to 13 weeks. Thereafter, the lumbar vertebrae and femora were analyzed.

OST + RAL treatment showed a favorable effect on structural and biomechanical bone parameters, demonstrating some advantages over RAL alone. RAL confirmed its antiresorptive effect on bone tissue without causing negative systemic effects. OST alone was less effective in protecting bone tissue. It increased osteoblast number, serum phosphorus, bone magnesium, and inner organ and uterus weight. The adverse effect of OST on bone magnesium level was attenuated when combined with RAL. Conversely, the combined treatment increased serum phosphorus and luteinizing hormone levels, decreased serum magnesium and calcium, and did not attenuate the organ and uterus weight increase observed after OST, raising safety concerns. These findings highlight the need for cautious evaluation of combination therapies and suggest that there is a need for alternative compounds with improved safety profiles for future osteoporosis treatments.

**Supplementary Information:**

The online version contains supplementary material available at 10.1007/s00223-025-01431-4.

## Introduction

Both estrogen and androgen hormones play critical roles in maintaining bone density in women. They help regulate the remodeling process by balancing bone resorption and formation, which is crucial for maintaining bone density and preventing bone loss that can lead to osteoporosis [1]. However, they are not widely prescribed because of potential negative side effects [2, 3]. Non-steroidal selective estrogen and androgen receptor modulators (SERMs and SARMs) with higher selectivity and fewer side effects may be a promising treatment for osteoporosis. SERM raloxifene (RAL) is a drug prescribed for the treatment of postmenopausal osteoporosis [4, 5]. RAL reduces the occurrence of vertebral fractures, but not non-vertebral fractures [6]. Ostarine (OST), also known as S-22, enobosarm and GTx-024 is a SARM [7]. Similar to SERMs, SARMs have been developed to stimulate bone and muscle, with presumably lower effects on other testosterone-dependent tissues. Unlike testosterones, SARMs do not contain steroid rings. There is evidence for the positive effects of OST on total lean body mass and physical function in patients with cancer-induced muscle wasting and elderly men [7–9]. Its significant effects on physical function have also been demonstrated in postmenopausal women [8]. In a previous study, we demonstrated a significant improvement in the cortical and trabecular bone after ostarine treatment in an estrogen-deficient rat model [10]. Furthermore, OST has a positive effect on early bone healing in ovariectomized rats [11]. Data regarding combined SARM and SERM therapies in osteoporosis are limited. Positive additive effects on the bones of ovariectomized rats by the application of a non-steroidal SARM (S-101479) in combination with RAL have been reported [12]. Our latest study showed an improvement in bone tissue following combined treatment with OST and RAL in an orchiectomized rat model for male osteoporosis [13]. For muscle tissue, OST showed advantages over RAL and their combined treatments for muscle of estrogen-deficient rats [14].

In the present study, we investigated the effects of combined treatment with OST and RAL in an ovariectomized rat model [15], applied as osteoporosis prophylaxis. We hypothesized that the bone anabolic effect of OST [16], combined with the antiresorptive effect of RAL [17], would effectively prevent bone loss under estrogen-deficient conditions. Data on muscle tissues and data on animal model as a part of this study have been reported recently [14].

## Materials and Methods

### General Procedures

All procedures were approved by the regional government (permission number: 33.9–42,502-04–14/1396, Oldenburg, Germany). The experiment was performed using three-month-old female Sprague Dawley rats (Janvier Labs, Le Genest-Saint-Isle, France). Three to four rats per cage were maintained according to German animal protection laws and fed a soy-free diet throughout the experiment (ssniff Special Diet, Soest, Germany).

Fifteen rats were left intact and represented the healthy control group (NON-OVX group, n = 15). Sixty rats were ovariectomized (OVX) and divided into four groups (n = 15 per group). The first group did not receive any specific therapy (OVX). The second group received OST added to the diet at a concentration of 8.6 g/kg food (OST group). The third group was fed chow supplemented with RAL (171.4 g/kg of food; RAL group). The fourth group was fed a diet containing both OST and RAL (OST + RAL group) at the same concentrations as the second and third groups [10]. Treatments were administered for 13 weeks. OST was bought from Shanghai Biochempartner Co., Ltd. (Shanghai, China), and RAL was obtained from Eli Lilly and Company (Evista®, Indianapolis, USA). Food intake and body weight (BW) were recorded weekly. The average daily doses, calculated based on food intake in the cage, were 11.07 ± 1.77 mg/kg BW for RAL and 0.55 ± 0.08 mg/kg BW for OST. The dosages were derived from human studies [18, 19] using the dose conversion method described by Nair and Jacob [20] (Suppl. Table 1).

Thirteen weeks after ovariectomy, all the rats were decapitated under CO_2_ anesthesia. Lumbar vertebrae, femora, serum samples, and uteri were collected for several analyses [21]. Bone structure was analyzed using micro-computed tomography (micro-CT) of the fourth lumbar vertebral body (L4) and right femur. The second lumbar vertebral body (L2) and left femur were used for ashing analysis. The third lumbar vertebral body (L3) and the left femur were analyzed biomechanically. The fifth lumbar vertebral body (L5) was analyzed histologically, and gene expression analysis was performed in the sixth lumbar vertebral body (L6). Bone samples were stored at − 20 °C until analysis. For gene expression analysis, the samples were stored at − 80 °C, whereas for histological analysis, bone samples were fixed in 4% buffered formalin for 1 week and then stored for a few weeks in 70% ethanol [13].

#### Micro‑Computed Tomography

L4 and the right femur were scanned using Quantum FX microcomputed tomography (micro-CT; Caliper Life Sciences, Hopkinton, MA, USA) at 70 kVp, 200 μA, 2-min exposure time, 360° rotation, 3600 projections, 20 × 20 mm^2^ field-of-view, 512-pixel matrix, and 40 × 40 × 40 μm^3^ effective voxel size. A phantom block with five known mineral densities was included in each scan [13, 21]. Bone samples were analyzed using the 3D OsteoAnalyze software (developed in our laboratory). A later version of this program is the Scry v6.0 software (Kuchel & Sautter UG, Bad Teinach-Zavelstein, Germany) [22]. In L4, the region of interest was the corpus vertebra, whereas in the femur, the femoral head was digitally cut in the transition zone from the collum femoris to trochanter major [21].

The following bone parameters were assessed using three-dimensional (3D) analysis according to the American Society for Bone and Mineral Research [23]: bone volume fraction (BV/TV, %), total BMD (Tt.BMD, g/cm^3^), bone volume (BV, mm^3^) cortical BMD (Ct.BMD, g/cm^3^), cortical volume (Ct.V, mm^3^), trabecular BMD (Tb.BMD, g/cm^3^), trabecular volume (Tb.V, mm^3^), soft tissue volume (St. V) [13].

The bone structure was analyzed using 2D images of micro-CT scans (n = 3 per bone sample) created using the 3D OsteoAnalyze program. Three images of sagittal cut L4 and the femoral head were analyzed using MetaMorph Basic Acquisition Software (Leica Mikrosysteme Vertrieb GmbH, Wetzlar, Germany). The collected bone data included the number of trabecular nodes (Tb.Nd) and trabecular thickness (Tb.Th). Trabecular bone area fraction (Tb.Ar/Tt.Ar, %) and cortical bone area fraction (Ct.Ar/Tt.Ar, %) were calculated from 2D images as the ratio of bone area (Tb.Ar or Ct.Ar) to the total area (Tt.Ar) of the corresponding bone regions [24, 25].

#### Biomechanical Assessment

Biomechanical tests of the L4 and left femur were performed according to a previously developed method using a Zwick testing machine (1446, Zwick, Ulm, Germany) [26, 27]. The thawed bones were fixed on the base on the machine and the stamp was lowered at a speed of 50 mm/min. The load was measured using an Xforce P load cell (Zwick) with a relative accuracy of 0.2–0.4% over the range of 2–500 N. The machine automatically stopped when the force dropped below 10 N. During the test, data were recorded every 0.1 mm using the testXpert software (Zwick). L4 was evaluated using a compression test. It was fixed to an aluminum base, and a loading stamp was positioned on the cranial aspect of the vertebral body. A vertical load was applied until the first fractures occurred, simulating physiological load conditions [13, 21]. The femur was assessed using a simulated fall-related loading setup. The femoral head was placed into a 4 mm well on the base and mid-shaft was stabilized using two lateral screws to prevent displacement. Load was applied vertically to the trochanteric region, inducing a localized fracture at the femoral neck [13, 21]. The maximum recorded force was defined as the ultimate load (N). Stiffness was determined as a slope of the linear portion of the force–displacement curve during elastic deformation. For L4, the cross-sectional area of the cranial plate and the height of the vertebral body, were measured using micro-CT slices. Ultimate stress was calculated by dividing ultimate force by the cross-sectional area (N/mm^2^ or MPa). Young's modulus (MPa) was obtained by multiplying stiffness by the height of the L4 and dividing by the cross-sectional area of L4. For the femur, the results were normalized using the cross-sectional moment of inertia (CSMI) and the section modulus of the femoral neck, since the femoral mechanical test represented bending rather than pure compression, as in L4 [28, 29]. CSMI was estimated by approximating the femoral neck cross-section as an ellipse, using two orthogonal diameters measured across the outer cortical (periosteal) contour on micro-CT slices ((π / 4 x 9major diameter/2)^3^ x (minor diameter/2)). The ultimate stress (MPa) was calculated by dividing the bending moment at the femoral neck by the section modulus. The bending moment was determined from the ultimate load and the femoral neck length (ultimate load x length/4). The section modulus was derived by dividing the CSMI by the half of the major diameter of the femoral neck. The apparent Young’s modulus (MPa) was calculated by normalizing stiffness to the femoral neck geometry, taking into account CSMI and femoral neck length ((stiffness x length^3^) / (48 × CSMI)) [29]**.**

#### Ashing

The L2, and left femur were ashed in a muffle oven at 750 °C for 2 h. Bone samples were weighed before and after ashing. The mineral content was determined by ash weight and expressed relative to the wet weight of each bone (%). Calcium and magnesium contents were assessed using an atomic absorption spectrometer (4100, PerkinElmer, Waltham, MA, USA), according to the European Committee for Standardization (CEN)[30]. Orthophosphate content was determined using a colorimetric method (spectral photometer DM4, Zeiss, Oberkochen, Germany) according to CEN [31]. The amount of minerals is shown as a percentage of the weight of the ash sample [21].

#### Gene Expression Analysis

Gene expression analysis was performed at the L6 using quantitative real-time polymerase chain reaction (qRT-PCR). Bone samples were homogenized using a microdismembrane (Sartorius, Goettingen, Germany). Thereafter, 50 mg bone powder was mixed with 500 µl TRIzol reagent (Thermo Fischer Scientific, WA, USA). The samples were incubated for 5 min at room temperature and further RNA extraction was performed according to the manufacturer’s protocol (TRIzol; Thermo Fischer Scientific) using chloroform and isopropanol treatments and ethanol washing. Finally, the RNA pellet was dissolved in 20 µL Rnase free water, measured by DeNovix DS-11 FX + System (DeNovix, NC, USA) and stored at − 80 °C for further analysis [14]. After extraction, the RNA (1000 ng) was reverse-transcribed using an iScript cDNA synthesis kit (Bio-Rad, Hercules, CA, USA). The expression levels of alkaline phosphatase (Alp), androgen receptor (Ar), estrogen receptor-α (Er-α), estrogen receptor-β (Er-β), receptor activator of nuclear factor κB ligand (Rankl), osteoprotegerin (Opg), and osteocalcin (Oc) were measured using SYBR green detection (QuantiTect SYBR Green PCR Kit, Qiagen, Venlo, The Netherlands) in an iCycler system (CFX96, Bio-Rad Laboratories, Hercules, CA, the USA). Ready-to-use primers from Qiagen were used and qRT-PCR was performed with 40 cycles and 55 °C annealing temperature. Gene expression was calculated using the 2^−ΔΔCT^ method [32]. The results are expressed relative to the gene expression levels in the NON-OVX group. β-2 microglobulin was taken as reference gene [21]. The OPG/RANKL ratio was calculated using Excel program (Microsoft, Redmond, USA).

#### Histomorphometrical Analysis

L5 samples stored in ethanol were embedded in Technovit 9100 New® (Heraeus Kulzer GmbH, Wehrheim, Germany) and cut longitudinally using a Leica microtome (RM 2165, Leica Instruments GmbH) at a thickness of 5 µm. Thereafter, the sections were deacrylated, stained with Toluidine Blue O (Merck, Darmstadt, Germany), and mounted with Eukitt (O. Kindler GmbH, Freiburg, Germany). The sections were digitalized using a digital camera (Leica DFC490) and zoom stereo microscope (Leica DMRXE, Bensheim, Germnay) and analyzed using the MetaMorph image analysis program (Leica, Bensheim, Germany). Three randomly chosen fields (0.1 mm^2^ within the histological section were used for the analyses. The following parameters were measured: osteoblast number per bone perimeter (N. Ob/B. Pm), osteoclast number per bone perimeter. (N. Oc/B. Pm), and the number of osteocytes per bone area (Ot/B. Ar) [13, 24].

#### Serum Analysis

Serum alkaline phosphatase (AP), calcium (Ca), magnesium (Mg), and phosphorus (P) analyses were conducted at the Department of Clinical Chemistry, University Medical Center, Goettingen, using commercial tests (Architect, Abbott, Wiesbaden, Germany) and an automated chemistry analyzer (Architect c16000 Analyzer, Abbott) [13]. Osteocalcin (OC) and the cross-linked C-telopeptide of type I collagen (CTX-I) were assessed using enzyme linked immunosorbent assay (EIA), rat-MID Osteocalcin EIA, and RatLaps™ (CTX-I) EIA, respectively (Immunodiagnostic Systems GmbH, Frankfurt am Main, Germany). Follicle-stimulating hormone (FSH) and luteinizing hormone (LH) levels were measured using an EIA kit for rats (Cloud-Clone Corp., Katy, Texas, USA) [21].

#### Statistical Analysis

Statistical analyses were performed using SAS program (version 9.1; SAS Institute, Cary, NC) and GraphPad Prism (version 5.04, GraphPad Software Inc. San Diego, USA). The effects of treatments were assessed using analysis of variance (ANOVA), with body weight (BW) included as a covariate, along with the interaction between treatments and BW. Since no significant interaction between treatments and BW was observed for any parameter, the final model included only treatment group and BW. Parameters for which BW was a significant covariate are presented as BW-adjusted least squares means (LSMeans) with standard error (SE). Parameters for which BW had no significant effect are presented as means with standard deviation (SD).

The normality of data distribution was tested using the Kolmogorov–Smirnov, D’Agostino–Pearson omnibus, and Shapiro–Wilk tests. Data were considered normally distributed if they passed at least one of these tests. For normally distributed variables, ANOVA followed by Tukey’s post hoc test was used to evaluate group differences (p < 0.05). For non-normally distributed variables, the Kruskal–Wallis test followed by Dunn’s multiple comparison test was applied (p < 0.05). Unpaired t-tests (p < 0.05) were used to compare average doses of OST and RAL. Correlation analyses between body weight and the weights of internal organs and uterus were also conducted.

## Results

### Animal Model

The average BW was higher in the OVX compared to the other groups (Suppl. Table 1). Treatment of OVX rats with OST did not change it, whereas RAL decreased it to the lowest level among the group. RAL + OST maintained average BW at the level of healthy NON-OVX group.

The average food intake [14] was also higher in the OVX and OST groups than in the other groups, whereas rats in the RAL group consumed less food on average than those in the OST + RAL group (Suppl. Table *1*). The calculated average doses of OST and RAL [14] were lower when the substances were applied alone than when used in combination (Suppl. Table 1).

The BW measured at the end of the study, which was taken as a covariate in the statistical analysis, was higher in the OVX group that that in the NON-OVX group and both groups treated with RAL (RAL and OST + RAL), with the highest BW observed in the OST group (Suppl. Fig. 1) [14]. BW had a significant impact on the weight of heart, liver, kidney and spleen, therefore, LSMeans were compared. OVX rats had lower liver and uterine weight and higher visceral fat weight compared to NON-OVX rats. In the OST treated rats, liver, heart, kidney and uterus weight was higher and visceral fat weight was lower than in the OVX untreated rats. RAL treatment did not affect organ weight in OVX rats, whereas the effect of OST + RAL was similar to that of OST alone (Suppl. Figure *1*). An exception was visceral fat weight, which was lower in the RAL and OST + RAL groups compared to the both OVX and OST groups (Suppl. Figure 1). Positive, highly significant correlations were observed between BW and the weight of organs and visceral fat (Suppl. Table 2). The correlation between BW and uterine weight was not significant (Suppl. Table 2).

#### Serum Analyses

Serum CTX-I level was higher, while calcium and magnesium levels were lower in the OVX rats than in the NON-OVX rats (Suppl. Table 1). In OST treated rats, AP activity and P levels were higher than in the OVX and NON-OVX rats. Under RAL treatment, P and FSH levels were higher than those in the NON-OVX rats. In the OST + RAL group, serum OC, Ca, and Mg levels were lower, whereas P and LH levels were higher compared to the OVX group [14]. AP and FSH levels were also higher in the OST + RAL group than that in the NON-OVX group (Suppl. Table 1).

#### Micro-CT Analyses

**Fig. 1:**
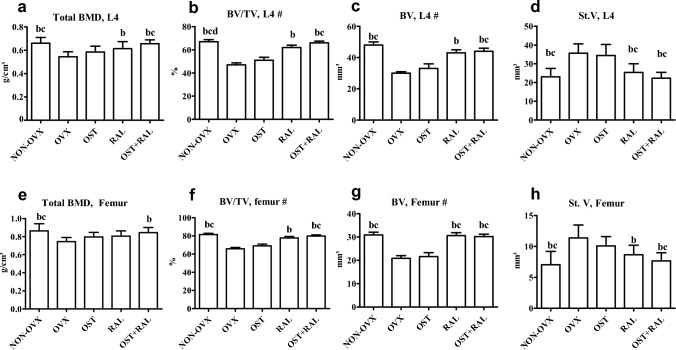
3D microCT of L4 (a-d) and femur (e–h) conducted in OVX rats either untreated or treated with ostarine (OST), raloxifene (RAL) or combined treatment (OST + RAL) and in NON-OVX rats. # Values are shown as BW adjusted least-squares means with SE where BW was a significant covariate; all other values are presented as means with SD. (b) differs from OVX, (c) differs from OST, (d) differs from RAL (p < 0.05, Tukey test)

In the OVX group, most 3D bone parameters in both the femur and L4 were lower compared to the NON-OVX group, while soft tissue volume (St.V) was higher (Fig. 1a–h, Table 1). OST treatment did not change 3D bone parameters in the OVX rats. Under RAL treatment, Ct.V, total BMD, BV/TV, BV in L4 and BV/TV and BV in the femur were higher than in OVX rats, whereas St.V was lower in both bone analyzed. The effect of OST + RAL on 3D bone parameters was similar to that of RAL alone, with additional increases in Tb.BMD and Tb.V in L4, as well as Tb.BMD and total BMD in the femur (Fig. 1a-h, Table 1).

**Table 1:** Ashing analysis of L3 and femur, gene expression in L6 and histomorphometry in L5 of OVX rats either treated with ostarine (OST), raloxifene (RAL) or combined treatment (OST + RAL) and NON-OVX rats

Parameters	NON-OVX		OVX			OST				RAL			OST + RAL	
*Micro-CT 3-D* *L4*														
Tb.BMD (g/cm^3^) #	0.78^bc^	0.004	0.76	0.004		0.76		0.006		0.78	0.005		0.78^bc^	0.004
Tb.V (mm^3^) #	35^b^	1	24	1		28		2		31	2		33^b^	1
Ct.BMD (g/cm3)	1.19	0.02	1.17^a^	0.02		1.16^a^		0.02		1.17	0.02		1.18	0.02
Ct.V (mm^3^) #	13^bc^	1	6	1		5		1		12^bc^	1		11^bc^	1
*Right femur*														
Tb.BMD (g/cm^3^) #	0.88^bc^	0.01	0.84	0.01		0.84		0.01		0.87	0.01		0.87^bc^	0.01
Tb.V (mm^3^) #	17	1	14	1		14		2		20	1		19	1
Ct.BMD (g/cm^3^)	1.22	0.05	1.23	0.06		1.23		0.05		1.22	0.05		1.23	0.05
Ct.V (mm^3^)	12	4	8^a^	2		10		3		9	3		11	3
*Ashing analysis*														
*L2* Mineral content (%)	39.6^b^	2.1	36.0		2.3		37.4		1.9	37.9	2.9		39.3^b^	1.6
*Left femur*														
Mineral content (%)	45.3^bc^	1.6	41.3 2.0				42.9		2.4	43.6^b^	2.1		45.2^bc^	1.0
Mg^+^ (%)	0.68	0.01	0.68		0.02		0.71*		0.01	0.68	0.02		0.67	0.02
Ca^2+^ (%) #	32.3^b^	0.6	35.6		0.6		35.5		0.9	32.9	0.6		32.9^b^	0.5
PO_4_^3−^ (%)	64.2	2.0	64.7		1.1		65.0		0.8	63.7	2.1		64.1	1.5
*Gene expression, L6*														
Opg	1.12	0.57	0.91		0.33		1.25		0.73	1.77^abe^	0.72		1.13	0.41
Rankl	1.05	0.36	1.12		0.59		0.68		0.38	0.94	0.64		1.22^c^	0.55
Opg/Rankl	1.18	0.72	0.90		0.24		2.01^be^		0.81	2.41^abe^		1.51	1.05	0.36
Oc	1.05	0.36	2.20^ae^		0.85		2.10^a^		0.47	2.30^ae^		1.36	1.30	0.46
Alp	1.04	0.29	0.95		0.38		1.18		0.56	1.54^e^	0.76		0.77	0.25
Er-α	1.29^bcd^	0.91	0.57		0.26		0.43		0.15	0.43	0.12		1.34^bcd^	0.49
Er-β	1.26	0.83	0.70		0.76		1.64		1.33	1.81		1.33	1.09	1.3
Ar	1.10^bcd^	0.52	0.66		0.28		0.57		0.20	0.67	0.20		1.13^bcd^	0.23
*Histomorphometry, L5*														
Ob/B.Pm (N/mm)	26	8	33		11		35^d^		14	24	13		31	11
Oc/B.Pm (N/mm)	3.0	0.6	3.2		0.4		3.4		1.1	3.1	0.9		3.5	1.0
Ot/B.Ar (N/mm^2^)	617	111	634		140		574		119	639	119		604	144

^#^ Values are shown as BW adjusted least-squares means with SE where BW was a significant covariate; all other values are presented as means with SD. *Differs from all other groups, ^**a**^differs vs. NON-OVX, ^**b**^vs. OVX, ^**c**^vs. OST, ^**d**^vs. RAL, ^**e**^vs. OST + RAL. (p < 0.05, Dunn’s test: mineral content in femur, Ob/b.Pm, Oc/B.Pm, Ct.V in L4 and Ct.BMD in femur, all gene expression data excluding OC; Tukey test: all other data)

**Fig. 2:**
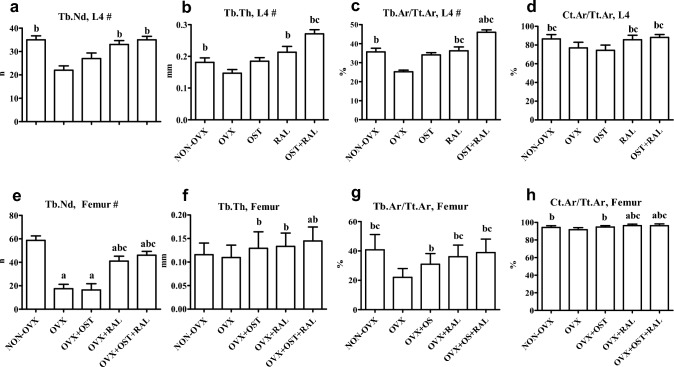
2D microCT of L4 (a-d) and femur (e–h) conducted in OVX rats either untreated or treated with ostarine (OST), raloxifene (RAL) or combined treatment (OST + RAL) and in NON-OVX rats. # Values are shown as BW adjusted least-squares means with SE where BW was a significant covariate; all other values are presented as means with SD. (a) differs from NON-OVX, (b) differs from OVX, (c) differs from OST (p < 0.05, Dunn’s test: Tb.Nd and Tb.Th in femur; Tukey test: all other data)

All 2D bone parameters, except Tb.Th, were lower in the OVX group than in the NON-OVX group (Fig. 2a-h). OST treatment increased Tb.Th, Tb.Ar/Tt.Ar, and Ct.Ar/Tt.Ar in the femur compared to that in the OVX group (Fig. 2f-h). Under RAL treatment, all 2D bone parameters in both L4 and femur were higher than in the OVX group. The effect of OST + RAL treatment was similar to that of RAL alone, all 2D bone parameters were significantly higher than those in the OVX group (Fig. 2.a-h). Tb.Th in the femur was higher in the OST + RAL group compared to the NON-OVX group, whereas Tb.Nd in the femur was higher, and Ct.Ar/Tt.Ar in the femur and Tb.Ar/Tt.Ar in L4 were larger in both the RAL and OST + RAL groups than in NON-OVX (Fig. 2.a-h).

#### Biomechanical Assessment

**Fig. 3:**
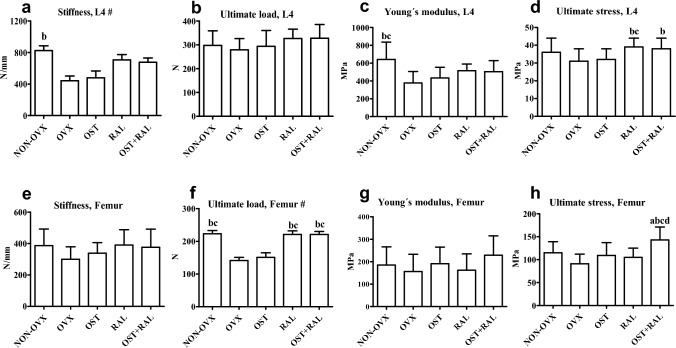
Biomechanical analysis of L4 (a-d) and femur (e–h) conducted in NON-OVX and OVX rats either untreated or treated with ostarine (OST), raloxifene (RAL) or combined treatment (OST + RAL). # Values are shown as BW adjusted least-squares means with SE where BW was a significant covariate; all other values are presented as means with SD. (a) differs from NON-OVX, (b) differs from OVX, (c) differs from OST, (d) differs from RAL (p < 0.05, Tukey test)

Stiffness and Young´s modulus in the L4 and the ultimate load in femora were significantly lower in the OVX group than in the NON-OVX group (Fig. 3a, c, f). Treatment with OST did not change the biomechanical and material bone parameters of OVX rats, which were also significantly lower compared to the NON-OVX, RAL, and OVX + RAL groups. Under RAL treatment, higher ultimate load in femur and ultimate stress in L4 were detected compared to the OVX group. OST + RAL treatment had a similar effect on the biomechanical properties as RAL alone (Fig. 3a-g). However, ultimate stress of the femur was higher in the OST + RAL group than in all other groups, including the RAL group (Fig. 3h).

There were no significant differences between the groups in most parameters of bone geometry used for data normalization. Specifically, the cross-sectional area (8.7 ± 0.7 mm^2^) and height (6.8 ± 0.3 mm) of L4 and the major diameter (2.9 ± 0.2 mm) and length (3.7 ± 0.4 mm) of femoral neck were consistent across the groups. However, the minor diameter of femoral neck was significantly smaller in the OST + RAL group (1.9 ± 0.2 mm) compared to the OVX and OST groups (both 2.1 ± 0.1 mm). In the NON-OVX and RAL groups, the minor diameter averaged 2.0 ± 0.1 mm.

#### Ashing

In the OVX group the mineral content of L2 and femur was lower than in the NON-OVX group, whereas Ca level was higher in femur (Table 1). OST did not affect mineral content, however Mg level was the highest in this group. Under RAL treatment, the mineral content of the femur was higher than in the OVX group. In the OST + RAL group, the mineral content of both the L2 vertebra and femur was higher, while calcium levels were lower compared to OVX rats (Table 1). The phosphate content (Table 1) and Ca^2+^/PO_4_^3−^ ratio (data not shown) in the femur were not significantly different between the groups.

#### Gene Expression

In the OVX group, Oc expression was higher, whereas Er-α and Ar gene expression were lower than in the NON-OVX group (Table 1). In OST-treated rats, the Opg/Rankl ratio was higher compared to the OVX group (Table 1). Under RAL treatment, the expression of Opg and the Opg/Rankl ratio were higher compared to the NON-OVX, OVX, and OST-RAL groups, while Alp and Oc expression were higher than in the OST + RAL group (Table 1). In the OST + RAL group, Oc expression was lower than in the OVX group, whereas Er-α and Ar expression were higher (Table 1).

#### Histomorphometrical Analysis

OVX did not change the histomorphometrical bone parameters (Table 1). The number of osteoblasts was higher in the OST group than in the RAL group, whereas the osteocyte and osteoclast parameters did not differ significantly between the experimental groups (Table 1).

## Discussion

Estrogen plays a key role in maintaining bone density by preventing excessive bone resorption, while testosterone, although present at lower levels, also contributes to bone formation and strength [1]. We previously demonstrated that therapeutic administration of OST improved bone parameters in an estrogen-deficient, osteoporotic rat model [10]. In the present study, however, OST was less effective in protecting bone when applied as osteoporosis prophylaxis immediately after ovariectomy. RAL confirmed its antiresorptive effect on bone tissue, and the combined OST and RAL treatment exhibited a comparable, yet slightly enhanced, bone-protective effect relative to RAL alone.

Single treatment with OST led solely to significant improvements in a few 2D parameters in the femur of OVX. The greater number of osteoblasts in OST-treated rats compared to the RAL group indicates a bone-forming response associated with SARM treatment [16, 17]. Additionally, the higher Opg/Rankl ratio in L6 and the elevated serum alkaline phosphatase levels, relative to the OVX group, are consistent with an anabolic effect of OST on bone tissue, reflecting enhanced osteoblastic activity [10, 33]. However, the biomechanical and material properties, as well as the 3D bone parameters of the femur and lumbar spine, were not affected by OST. Thus, the effect of OST was less pronounced than that in a previous study [10], probably because OST was administered as prophylactic treatment in the present study. The OST group exhibited the highest Mg content in the femur among all experimental groups, suggesting a potential role of SARMs in modulating mineral composition of bone tissue. This finding aligns with our recent results in orchiectomized male rats, where higher enhanced magnesium levels were also observed following 16 weeks of treatment with OST or testosterone [13, 34]. Dietary Mg deficiency affects bone tissue and can lead to osteoporosis [35, 36]. However, elevated levels of Mg in bone inhibit the formation of hydroxyapatite crystals by competing with Ca and binding to pyrophosphate to form insoluble salts that resist enzymatic degradation [37, 38]. Furthermore, the serum concentration of phosphorus was elevated by OST treatment in the present study. A similar effect was observed in our previous studies when OST was applied at the same dose to orchiectomized male rats for 16 weeks and to ovariectomized rats after a short application for 5 weeks [10, 34]. The bone-derived hormones fibroblast growth factor 23, parathyroid hormone, and vitamin D regulate phosphate concentration in the blood [39]. Whether these factors are affected by OST should be investigated in future studies, as hyperphosphatemia impairs osteoblast function, leading to bone loss and osteoporosis and is associated with vascular calcification and cardiovascular mortality [40–42].

Although the average body weight of the OST group was comparable to that of the untreated ovariectomized rats, it was the highest among the groups when measured at the end of the study. Accordingly, final body weight was included as a covariate to control for its potential confounding effects in the analysis. Heart, liver, and kidney weights were significantly higher in the OST group compared to the OVX group, whereas spleen and lung weights were unaffected by OST treatment. In this study, a strong positive correlation was found between body weight and the weights of all organs and visceral fat, consistent with previous findings in rats [13]. SARMs have also been reported to induce liver and kidney injuries and may increase the risk of cardiovascular disease [43, 44]. However, the low visceral fat weight observed under OST treatment, together with the highest muscle weight in this group [14], may indicate a favorable effect of OST on the musculoskeletal system in the ovariectomized rat model. However, the uterotrophic effect of OST could be an important safety limitation owing to the potential risk of cancer. Increased uterine weight appears to be a common problem with SARMS, as has been shown for several SARMs, including OST [45–48].

RAL treatment was associated with improvements in multiple 2D bone parameters, as well as several biomechanical, material and 3D bone characteristics compared to the untreated OVX rats. CTX-I, a sensitive indicator of bone loss, was maintained at levels similar to those in healthy NON-OVX rats. These findings, together with higher mineral content, greater Opg gene expression and Opg/Rankl ratio, and lower soft tissue volume, confirm the bone-protective effect of RAL. As an approved anti-osteoporotic drug for postmenopausal osteoporosis [4], our study further supports its protective role on bone in estrogen-deficient rats.

The present study demonstrated that RAL treatment successfully prevented weight gain that typically occurs after ovariectomy in rats [49] and also effectively maintained the weight of all the organs and visceral fat at the level of healthy NON-OVX rats. These parameters were positively correlated with body weight and were comparable to those measured in NON-OVX healthy rats. RAL prevents increase in abdominal fat and body weight in postmenopausal women and has shown similar effects in ovariectomized rat models [50, 51]. The administration of RAL has been shown to have an appetite-suppressing effect [52], which may have resulted in a reduction in food consumption and a consequent reduction in the body weight of the rats in our study. In addition, RAL did not affect the uterine weight after OVX, which is in agreement with previous studies and confirms its selectivity [50, 53]. Furthermore, LH and FSH levels in the RAL group were comparable to those in the OVX group, suggesting that RAL did not significantly affect the hypothalamic-pituitary–gonadal axis under estrogen-deficient conditions, which is consistent with findings observed in postmenopausal women [54]. However, FSH levels remained higher than those in the NON-OVX group, consistent with the known upregulation of FSH under conditions of estrogen deficiency [55].

The combined treatment with OST and RAL demonstrated favorable effects on most bone parameters, similar to those observed with RAL treatment alone. However, some bone parameters were more strongly affected by OST + RAL treatment than by RAL alone, e.g. mineral content in L2, Tb.BMD and Tb.V in L4 as well as Tb.BMD, Tt.BMD and ultimate stress in femur. Furthermore, side effects associated with OST monotherapy appeared less pronounced with the combined treatment. In the OST + RAL group, femoral Mg content, which was high in the OST group, was observed at lower levels, whereas expression of androgen and estrogen receptors was found at higher levels than in the OST group. Another SARM (S-101479), applied in combination with RAL, demonstrated a more pronounced effect on bone parameters than single compounds in estrogen-deficient female rats [12]. In an orchiectomized rat model of aged male osteoporosis, the combination of osteoanabolic OST, which stimulates osteoblasts and increases the mineralization rate [16, 17], with antiresorptive RAL, which diminishes osteoclast activity and stabilizes the mineralization rate of bone [16, 17], showed strong positive effect on bone tissue [13]. Sex hormones play a critical role in maintaining bone mass and strength in both men and women [1]. However, the combination of RAL and OST, which act selectively through sex hormone receptors [56], appears to offer only a modest advantage over RAL treatment alone in our rat model.

Food intake, body weight, and heart and visceral fat weights under OST + RAL treatment were maintained at the levels similar to those of healthy NON-OVX rats, consistent with previous findings in male orchiectomized rats [13]. However, the combined treatment did not mitigate the effects of OST on liver, kidney, or uterus weights, which remained at high levels similar to those observed in the OST group. Additionally, compared to the OVX group, the combined treatment was associated with lower serum levels of Ca, Mg, and osteocalcin, and higher levels of P and LH. FSH levels were higher than those in the NON-OVX rats, reflecting the persistent estrogen deficiency´s influence on the hypothalamic-pituitary–gonadal axis [55]. In orchiectomized male rats, a similar treatment also led to reduced serum osteocalcin and increased P levels [13]. In contrast, AP and Ca levels were elevated, and FSH and LH levels remained unchanged following OST + RAL treatment in orchiectomized rats [13]. Whether these discrepancies are attributable to differences in sex, age (6 vs. 13 months), or treatment duration (13 vs. 16 weeks) remains unclear. Whether these discrepancies are due to differences in sex, age (6 vs. 13 months), or treatment duration (13 vs. 16 weeks) between our study and the other [13] remains unclear. Nevertheless, due to their physiological relevance, these parameters warrant further investigation. The known adverse uterine effect of OST, likely related to the high density of androgen receptors in the uterus [57], was not attenuated by co-administration of RAL, which may represent a limitation of the combined treatment approach.

In the present study, ovariectomy was confirmed by higher body, organ and visceral fat weights, uterine atrophy, impaired bone structural and biomechanical parameters, and lower bone mineral content in the OVX groups compared to the NON-OVX group. Following OVX, serum CTX-I level was greater, whereas serum calcium and magnesium levels, as well as the expression of sex hormone receptors (ER-α and AR) in bone were lower. These observations are characteristic of the OVX rat model which is widely applied due to its reproducible bone-loss responses and practical advantages [15, 58]. However, a limitation of this study is the age of the animals at the time of ovariectomy, as well as the absence of a baseline control group. Although bone growth slows significantly after skeletal maturation [58], the animals continued to grow during the experimental period in our study [14], which may have led to an overestimation of treatment efficacy. For more translational relevance to postmenopausal or age-related bone loss, the use of 6-month-old or older rats is recommended [15, 58, 59]. Another limitation is that the animals were not pair-fed and had ad libitum access to food, leading to variability in nutritional intake and body mass. To account for these differences, body weight was considered as a covariate in the analysis of bone parameters.

In conclusion, our data showed that the combined treatment with OST and RAL provided overall beneficial effects on bone tissue similar to those of RAL alone, while surpassing RAL in several bone parameters. RAL confirmed its antiosteoporotic effect on bone without causing negative systemic effects in estrogen-deficient rat model. Although OST treatment had a limited effect on bone, it significantly reduced visceral fat weight and demonstrated a strong positive effect on skeletal muscle tissue in these rats, as reported previously [14]. However, OST also induced some unfavorable systemic effects. While combining OST with RAL mitigated certain negative outcomes, it also introduced additional side effects and did not reduce all safety concerns associated with OST alone. These findings represent a limitation of both OST and combined OST + RAL treatments due to safety concerns. Nevertheless, combinations of other SERMs and SARMs with higher selectivity and fewer side effects than those examined in this study may be more suitable for future osteoporosis treatment strategies.

## Supplementary Information

Below is the link to the electronic supplementary material.

Supplementary file1 (PPTX 173 KB)

Supplementary file2 (DOCX 19 KB)

Supplementary file3 (DOCX 15 KB)
