# Supplementary material for: Effects of Combined Treatment With Selective Androgen and Estrogen Receptor Modulators Ostarine and Raloxifen on Bone Tissue In Ovariectomized Rats
Source: Calcif Tissue Int. 2025 Oct 24;116(1):133. doi: 10.1007/s00223-025-01431-4 (PMC12552363; doi:10.1007/s00223-025-01431-4)
Supplement: Supplementary file 1 — Supplementary file1 (PPTX 173 KB) [file 223_2025_1431_MOESM1_ESM.pptx]

## Slide 1
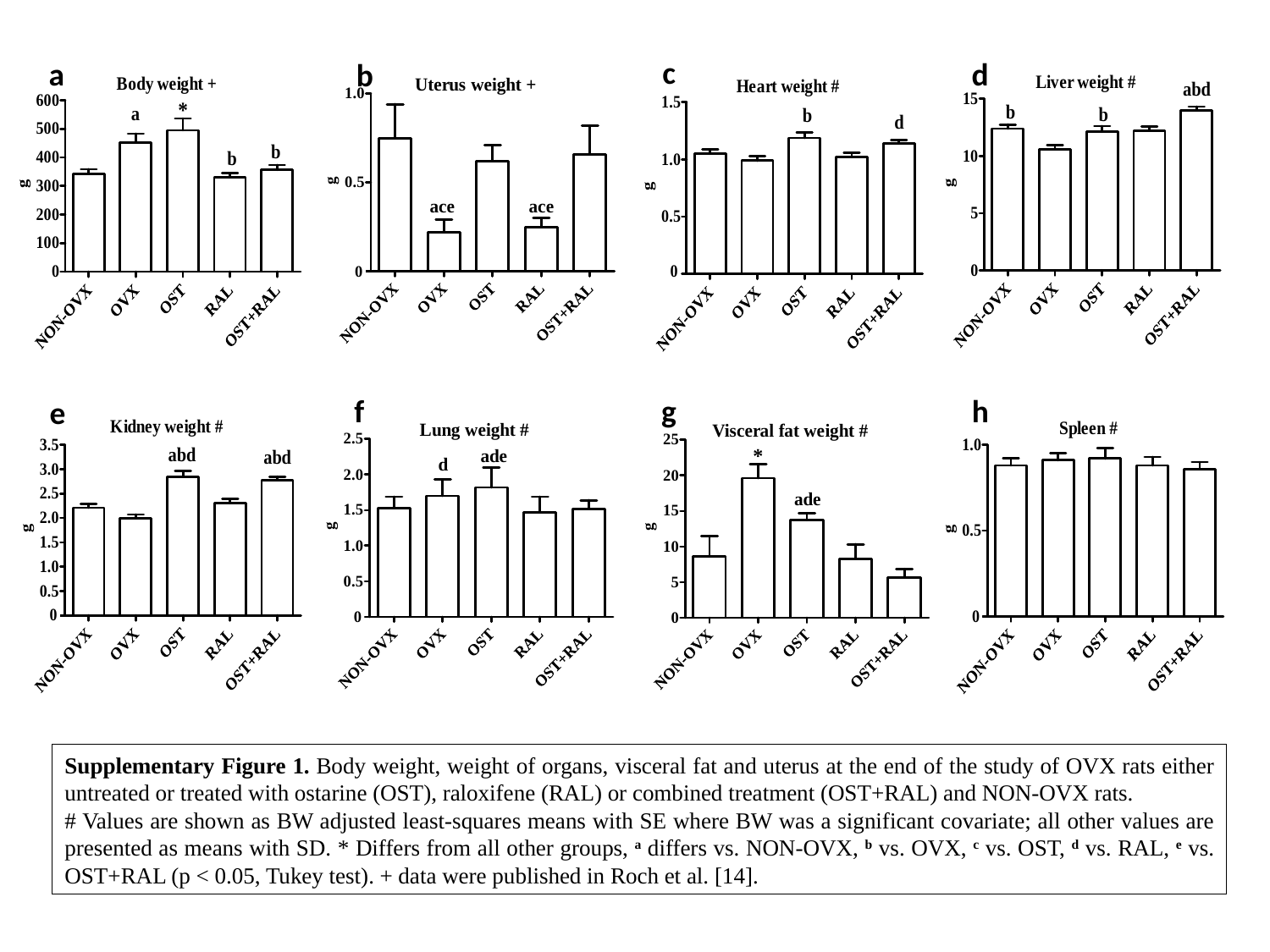

c
d
a
b
f
g
h
e
Supplementary Figure 1. Body weight, weight of organs, visceral fat and uterus at the end of the study of OVX rats either untreated or treated with ostarine (OST), raloxifene (RAL) or combined treatment (OST+RAL) and NON-OVX rats.
# Values are shown as BW adjusted least-squares means with SE where BW was a significant covariate; all other values are presented as means with SD. * Differs from all other groups, a differs vs. NON-OVX, b vs. OVX, c vs. OST, d vs. RAL, e vs. OST+RAL (p < 0.05, Tukey test). + data were published in Roch et al. [14].
