# Supplementary material for: Effects of Combined Treatment With Selective Androgen and Estrogen Receptor Modulators Ostarine and Raloxifen on Bone Tissue In Ovariectomized Rats
Source: Calcif Tissue Int. 2025 Oct 24;116(1):133. doi: 10.1007/s00223-025-01431-4 (PMC12552363; doi:10.1007/s00223-025-01431-4)
Supplement: Supplementary file 2 — Supplementary file2 (DOCX 19 KB) [file 223_2025_1431_MOESM2_ESM.docx]

**Supplementary Table 1**. Food intake, body weight (BW) and dose averaged over the weeks and serum analysis of OVX rats either untreated or treated with ostarine (OST), raloxifene (RAL) or combined treatment (OST+RAL) and NON-OVX rats

| Parameters | NON-OVX | | OVX | | OST | | RAL | | | OST+RAL | |
| --- | --- | --- | --- | --- | --- | --- | --- | --- | --- | --- | --- |
| Average food intake (g/rat/day)+ | 21 | 3 | 25**^ade^** | 4 | 26**^ade^** | 3 | 19**^e^**  4 | | | 22 | 5 |
| Average dose, (mg/kg BW/day)+ | | |  |  | 0.52**^e^** | 0.08 | 10.7**^e^** 2.1 | | OST: 0.58 0.11  RAL: 11.6 2.2 | | |
| Average BW | 319 | 25 | 401**^ae^** | 53 | 423**^ae^** | 68 | 308* | 21 | | 326 | 28 |
| ***Serum analysis*** |  |  |  |  |  |  |  |  | |  |  |
| AP (U/l) | 135 | 38 | 158 | 47 | 220**^ab^** | 73 | 180 | 36 | | 190**^a^** | 49 |
| OC (ng/ml) | 334 | 112 | 384 | 62 | 357 | 103 | 310 124 | | | 205**^bc^** | 41 |
| CTX-I (ng/ml) | 18.5 | 3.6 | 27.4**^a^** | 3.6 | 27.1**^a^** | 8.1 | 21.0 | 5.5 | | 21.7 | 6.3 |
| Ca (mmol/l)+ | 2.56* | 0.11 | 2.45 | 0.09 | 2.38 | 0.08 | 2.37 | 0.06 | | 2.36**^b^** | 0.06 |
| P (mmol/l)+ | 1.60 | 0.22 | 1.71 | 0.20 | 2.02**^ab^** | 0.18 | 1.90**^a^** | 0.23 | | 2.25***** 0.23 | |
| Mg (mmol/l)+ | 0.90* | 0.07 | 0.82 | 0.06 | 0.78 | 0.05 | 0.77 | 0.05 | | 0.76**^b^** | 0.05 |
| LH (pg/ml) | 276 | 44 | 302 | 66 | 369 | 70 | 335 | 111 | | 457**^ab^** | 104 |
| FSH (ng/ml) | 16.4 | 3.0 | 20.1 | 3.0 | 20.3 | 2.8 | 23.3**^a^** | 2.8 | | 25.2**^a^** | 4.4 |

* Means are different from all other groups, **^a^** differs vs. NON-OVX, **^b^** vs. OVX, **^c^** vs. OST, **^d^** vs. RAL, **^e^** vs. OST+RAL (p < 0.05 Tukey test). + data were published in Roch et al. [14]
