# Supplementary material for: Effects of Combined Treatment With Selective Androgen and Estrogen Receptor Modulators Ostarine and Raloxifen on Bone Tissue In Ovariectomized Rats
Source: Calcif Tissue Int. 2025 Oct 24;116(1):133. doi: 10.1007/s00223-025-01431-4 (PMC12552363; doi:10.1007/s00223-025-01431-4)
Supplement: Supplementary file 3 — Supplementary file3 (DOCX 15 KB) [file 223_2025_1431_MOESM3_ESM.docx]

**Supplementary Table 2.** Correlations of weight (W) of internal organs and uterus with body weight (BW) assessed by Pearson´s coefficient (r), two-tailed P-value.

| Correlations (n=73) | Pearson r | P |
| --- | --- | --- |
| Heart W - BW | 0.775 | <0.0001 |
| Liver W - BW | 0.830 | <0.0001 |
| Kidney W – BW | 0.551 | <0.0001 |
| Spleen W – BW | 0.776 | <0.0001 |
| Lung W - BW | 0.537 | <0.0001 |
| Visceral fat W – BW | 0.686 | <0.0001 |
| Uterus W – BW | -0.04 | 0.709 |
